# Complication development trajectories for patients with type 2 diabetes mellitus: evidence from a five-million retrospective cohort study

**DOI:** 10.3389/frhs.2025.1699513

**Published:** 2025-11-25

**Authors:** Haoqing Tang, Mingyue Li, Xiaokang Ji, Qingbo Zhao, Yongchao Wang, Yifu Zhao, Qing Wang, Fuzhong Xue, Xiaoyun Liu

**Affiliations:** 1 Department of Health Policy and Management, School of Public Health, Peking University, Beijing, China; 2 China Center for Health Development Studies, Peking University, Beijing, China; 3 School of Public Health, Shandong University, Jinan, Shandong, China

**Keywords:** type 2 diabetes mellitus, complications, trajectories, cohort study, PHC

## Abstract

**Background:**

Type 2 diabetes mellitus (T2DM) is a growing health burden in China. High complication rates contribute to increased morbidity, mortality, and costs. However, evidence is limited regarding how these complications develop and cluster over time in real-world settings, which this study examined.

**Methods:**

This retrospective cohort study used big data from the Cheeloo Lifespan Electronic Health Research Data Library, comprising data from over 5 million individuals in Shandong Province, China, to investigate the trajectories, onset timing and key risk factors of T2DM-related complications.

**Results:**

The prevalence of T2DM-related complications increased from 30.4% in 2013 to 53.1% in 2023. The median time from diagnosis to the first complication was 7.5 years. Ophthalmic, neurological, and circulatory complications were among the most common and showed the largest relative increases in prevalence over the study period. Complication profiles exacerbated over time: most patients developed multiple complications by Year 9. Frequent follow-up visits (≥4 times/year) and using primary health care (PHC) services were significantly associated with a reduced risk of complications, whereas being unmarried, being overweight, being obese, alcohol use, and poor medication adherence were significantly associated with a higher risk.

**Conclusion:**

Patients with T2DM in China face a substantial and growing burden of complications, with most developing multiple complications within ten years of diagnosis. Follow-up visits, the use of PHC services, and regular medication adherence are potential protective factors to prevent or delay the development of complication. These findings highlight the importance of integrated, community-based, and personalized management strategies to improve outcomes in T2DM populations.

## Introduction

Diabetes is a leading chronic condition that poses a growing threat to global health. In 2022, an estimated 828 million adults were diagnosed with diabetes, with a substantial increase from 630 million in 1990 (1). Between 1990 and 2022, the global prevalence of diabetes increased from 7% to 14%, with the sharpest increase observed in low- and middle-income countries (LMIC) (2). China has the highest global diabetes burden (3). In 2021, approximately 141 million Chinese adults had diabetes, accounting for over one-quarter of the global cases, with projections exceeding 174 million by 2045 (4). The national prevalence reached 13.0% in 2021, with an average annual per capita diabetes-related health expenditure of over USD 1,100 (5).

The growing prevalence of type 2 diabetes mellitus (T2DM) is particularly concerning owing to its chronic nature and the high burden of associated complications, including cardiovascular disease, nephropathy, neuropathy, and retinopathy (6). Furthermore, patients with T2DM-related complications experience higher rates of hospitalization, longer hospital stays, and poorer quality of life than those without complications (7). In 2019, 76.03 thousand deaths and 2.13 million disability-adjusted life-years (DALYs) were attributed to diabetes-related chronic kidney disease in China, of which T2DM accounted for 83.32% and 77.0% respectively (8).

Although T2DM-related complications frequently co-occur and evolve over time, systematic investigations of their longitudinal trajectories remain limited, particularly in LMIC settings (9, 10). Over the past decade, several large LMIC-based cohort studies have expanded our understanding of diabetes complications. For example, the Center for cArdiometabolic Risk Reduction in South Asia (CARRS) cohort and its follow-up analyses have provided robust evidence on renal function decline and cardiometabolic risk among South Asian adults with T2DM (11, 12). Likewise, the PURE study across 21 countries and the LANDMARC trial in India have yielded valuable longitudinal insights into diabetes management and cardiovascular outcomes in LMIC contexts (13, 14). More recently, Wu et al. (2025) identified and visualized the temporal trajectories of traditional and non-traditional diabetes complications in Asia, highlighting the increasing complexity of multi-organ involvement among diabetic populations (15).

Nevertheless, despite these advances, most studies in LMICs have focused on single organ systems—such as renal, ophthalmic, or cardiovascular complications—or on isolated endpoints like hospitalization or mortality. Few have comprehensively examined how multiple T2DM-related complications emerge, cluster, and progress concurrently within an integrated, population-based framework (16, 17). Moreover, existing studies from high-income countries tend to face limitations in sample size, follow-up duration, and cross-system data integration (18–21) For instance, the Hong Kong Diabetes Database reported declining cardiovascular–renal complications between 2000 and 2012, offering valuable insight, yet it remains confined to a single high-resource urban setting (22). In mainland China, population-based evidence remains scarce on how multiple complications evolve, interact, and accumulate over time.

To address the growing burden of T2DM and hypertension, China launched the National Essential Public Health Service Package (NEPHSP) in 2009 (23). This nationwide initiative established a core set of public health services to be delivered at the primary health care (PHC) level, making them universally accessible. In addition to prioritizing the management of hypertension and T2DM, the NEPHSP covers the establishment of individual health records and health education (23). For residents aged 35 years and older, the program provides routine chronic disease management services, including screening, quarterly follow-ups, disease monitoring, hospital referrals, and annual health examinations, to promote early detection and continuous care (24). However, despite the nationwide implementation of the NEPHSP and its emphasis on regular follow-up and chronic disease management, empirical evidence regarding the extent to which these follow-up visits prevent or delay diabetes-related complications remains limited. Strengthening the understanding of how such complications evolve—from the initial diagnosis to the onset of single or multiple conditions—is therefore essential for developing patient-centered care strategies and optimizing the allocation of healthcare resources.

To fill these gaps, this study used a large-scale, integrated population-based dataset from Shandong Province to investigate how T2DM-related complications develop over time. By integrating multi-organ complication data within a real-world cohort, it allows for the depiction of ten-year longitudinal trajectories and the identification of behavioral and health service factors influencing these pathways. Specifically, it examined (1) the time from diagnosis to complication onset, (2) the longitudinal trajectories of the most common complication combinations over ten years, and (3) the association between behavioral and health service factors—including follow-up visits and use of primary healthcare—and the risk of developing diabetes-related complications.

## Methods

### Study design

This study conducted a retrospective cohort analysis leveraging longitudinal electronic health record data to examine the progression of T2DM-related complications, including the median time from diagnosis to complication onset, trajectories of the most common complication combinations, ten-year prevalence trends of individual complications, and key factors associated with complication occurrence. In addition, a repeated cross-sectional analysis was used to describe annual prevalence trends of T2DM comorbidities and related complications.

### Data source

This study utilized data from Cheeloo LEAD, formerly known as the Shandong Multicenter Healthcare Big Data Platform (25, 26). Cheeloo LEAD is a large-scale, population-based database constructed using a three-stage cluster random sampling method. Specifically, 4,912,928 individuals were sampled from 39 of 136 counties (districts) in Shandong Province, China, with a total population of approximately 101 million. The database integrates 149 structured data tables spanning the full life course of residents and covers health records, basic public health services, electronic medical records, outpatient medical records, health examinations, disease surveillance, medical insurance claims, death records, and environmental health data. Supplementary Figure S1 and previously published studies provide further details on the study population's design, sampling procedures, and demographic characteristics. Additional information about the Cheeloo LEAD database is available in the published descriptions and related materials (27–29). This study was approved by the ethics committee of Shandong University (ISTEC-SPH-SDU-20250601). The need for written informed consent was waived by the ethics committee of Shandong University, due to the retrospective nature of the study.

Electronic medical records and national basic public health service follow-up records were extracted for the sampled residents, and individual identification numbers were used as indices to merge information from the two datasets. To ensure the completeness of diagnostic information, this study included individuals diagnosed with T2DM who had at least two hospitalization between January 1, 2013, and December 31, 2023, as outpatient records often lacked detailed or standardized diagnostic data. To define follow-up time in this study, we set the baseline (index date) as the date of the first hospitalization during which a T2DM diagnosis was recorded, occurring between January 1, 2013, and December 31, 2023. Subsequent medical visits, hospitalizations, and complication diagnoses were tracked continuously through electronic health records until the end of the observation period, death, or loss to follow-up, whichever occurred first. Although electronic records were available for the entire period, follow-up time was calculated individually for each patient starting from their index date, ensuring a longitudinal assessment of complication development.

The samples were screened based on the International Statistical Classification of Diseases and Related Health Problems, 10th Revision (ICD-10) codes. Type 2 diabetes mellitus (T2DM) was identified using codes E11 and E14, as confirmed by the referring physician. All complication diagnoses were likewise confirmed by attending physicians and recorded in the electronic medical records. To address repeated records of the same complication, we only retained the first occurrence of each complication per participant during the follow-up period. Once a complication was identified, subsequent records of the same condition were excluded from further analysis to avoid duplication. Follow-up did not terminate upon the first occurrence of any complication; instead, participants were followed continuously to allow for the identification of multiple and sequential complications over time. After excluding individuals without a diabetes diagnosis and records missing key demographic or diagnostic information (age, sex, ID, or ICD codes), the final analytical sample consisted of 122,236 individuals; partial missingness in non-essential variables was handled using available-case analysis.

The primary outcome variable was T2DM-related complications, which were identified using ICD-10 subcodes and diagnostic modifiers under the primary codes E11 and E14. Each complication type was assigned a corresponding Charlson Comorbidity Index (CCI) score (Supplementary Table S1). The CCI, a widely used tool for quantifying the comorbidity burden, assigns weighted scores to chronic conditions based on mortality risk, disease severity, and expected healthcare resource utilization. The CCI weights were assigned in accordance with the original scoring algorithm developed by Charlson (30, 31). Comorbidity was defined as the coexistence of T2DM with one or more non-communicable diseases (NCDs) (32, 33). Comorbidities in T2DM included complications, typically resulting from T2DM-related pathophysiological changes over time, and other NCDs that are not necessarily caused by T2DM. The data for all outcomes in this study were obtained from audited clinical diagnosis records, and professional clinicians made disease diagnoses through imaging and ultrasonography.

Variables that may be associated with the occurrence of T2DM-related complications were evaluated, with a primary focus on two key independent variables: follow-up times and healthcare institution preference. In defining “complete follow-up,” we adopted the ≥4 visits/year cutoff, consistent with national practice: the National Basic Public Health Service Specification (Third Edition) explicitly includes follow-up and evaluation for T2DM patients as part of essential public health services, and implementation guidelines commonly require that confirmed T2DM patients receive at least 4 in-person follow-ups annually along with 4 free fasting blood glucose tests (34). In this study, follow-up visits referred to management activities conducted by township hospitals, village clinics, or community health centers, including outpatient consultations, telephone tracking, home visits, and annual health examinations, with a minimum of four in-person visits per year as stipulated by NEPHSP.

Additional covariates included age (categorized as 45–54, 55–64, 65–74, and 75 and above), sex (male, female), marital status (married or partnered, unmarried, and others), residence status (rural, urban), health insurance type [Urban Employee Basic Medical Insurance [UEBMI], Urban and Rural Resident Basic Medical Insurance [URRBMI], and others], smoking status, alcohol consumption, body mass index (BMI), and medication adherence. Medication adherence was determined based on the National Basic Public Health Service Specification (Third Edition), which classifies adherence as “regular” (taking medication as prescribed), “intermittent” (partial adherence), and “no medication” (34). Participants with “regular” were classified as regularly taking medication, while those with “intermittent” or “no medication” were classified as irregular medication use. Adherence status was assessed and recorded by qualified clinical physicians during follow-up visits. To account for baseline health status and disease burden, the total CCI score was included as a control variable in all models.

### Statistical analyses

This study analyzed the trends in comorbidities and complications among individuals with T2DM from 2013 to 2023. We first analyzed the prevalence of T2DM comorbidities and T2DM-related complications among the samples per year.

Subsequently, we conducted a retrospective cohort study to examine the progression of T2DM-related complications. Based on the individuals entering the cohort, baseline (Year 0) was defined as the date of each participant's first hospitalization record during the study period (in order to obtain an accurate diagnosis of complications). Years 1–10 represented the subsequent one- to ten-year periods following the baseline. The follow-up duration was calculated from the date of baseline hospitalization to the date of the last available follow-up record. The follow-up frequency and total CCI score were defined as the average number of follow-up visits and the average CCI score per year, respectively, before the onset of T2DM-related complications.

Descriptive analyses were used to report the characteristics of the study sample and the prevalence of T2DM-related complications. In addition, the median duration from the initial diagnosis of T2DM to the onset of various complications was calculated, and the diagnosis dates were extracted from the Basic Public Health Services database. The temporal patterns of complication burden were further examined by analyzing the trajectories of T2DM-related complication combinations over time, as well as the ten-year prevalence trajectories of individual complication types.

We used a Cox proportional hazards regression model to identify factors associated with the occurrence of T2DM-related complications. This method is well suited for time-to-event data and allows hazard ratios (HRs) estimations to quantify the impact of various demographic, behavioral, and healthcare-related covariates on the risk of developing complications over time. The occurrence of T2DM-related complications and the survival status at each follow-up were recorded. Survival curves for different factors were plotted using the Kaplan–Meier method and compared using the log-rank test. The results were reported as HRs and 95% confidence intervals (CIs). The proportional hazards (PH) assumption was evaluated using Schoenfeld residuals for each model. No major violations were detected, indicating that the PH assumption held for the included covariates.

All statistical analyses and plotting were performed using R (version 4.3.1, packages: tidyverse, survival, survminer and ggplot2; R Core Team, Vienna, Austria) (35). Statistical significance was determined as a two-sided *P*-value <0.05.

## Results

### Demographic characteristics

Table 1 presents the demographic characteristics of patients diagnosed with T2DM from 2013 to 2023. The sample size increased significantly, from 8,516 in 2013 to a peak of 48,323 in 2022, then decreased to 17,565 in 2023. The mean age consistently increased across the study period, from 64.4 years (±9.8) in 2013 to 68.8 years (±9.8) in 2023. The proportion of older adults (≥75 years) notably increased from 16.9% in 2013 to 28.6% in 2023. The sex distribution remained relatively balanced, ranging from 49.3% to 53.0% male across the years. A high proportion of patients were married or partnered throughout the period, consistently exceeding 89%. Urban residency was predominant, accounting for 67.6%–79.6% of the sample. Approximately half of the patients consistently held UEBMI throughout the study period, whereas the proportion enrolled in URRBMI steadily increased, reaching 42.0% by 2023. Supplementary Table S2 summarizes the demographic characteristics of the patients diagnosed with T2DM at baseline from 2013 to 2023.

**Table 1:** Demographic characteristics of patients with T2DM from 2013 to 2023 [*n* (%)/mean (sd)].

Variables	Demographic characteristics										
	2013	2014	2015	2016	2017	2018	2019	2020	2021	2022	2023
Total	8,516	15,108	18,193	24,042	31,838	40,711	45,763	43,780	48,428	48,323	17,565
Age (years)	64.4 (9.8)	64.5 (10.0)	65.1 (10.0)	65.4 (10.2)	65.8 (10.1)	66.2 (10.1)	66.7 (10.0)	67.1 (10.0)	67.4 (9.9)	68.0 (9.9)	68.8 (9.8)
45–54	1,518 (17.8%)	2,732 (18.1%)	3,072 (16.9%)	4,107 (17.1%)	5,181 (16.3%)	5,825 (14.3%)	5,791 (12.7%)	5,011 (11.4%)	5,277 (10.9%)	4,824 (10.0%)	1,495 (8.5%)
55–64	2,863 (33.6%)	4,945 (32.7%)	5,817 (32.0%)	7,231 (30.1%)	9,229 (29.0%)	12,019 (29.5%)	13,108 (28.6%)	12,257 (28.0%)	13,188 (27.2%)	12,448 (25.8%)	4,304 (24.5%)
65–74	2,697 (31.7%)	4,780 (31.6%)	5,789 (31.8%)	7,731 (32.2%)	10,517 (33.0%)	13,796 (33.9%)	16,419 (35.9%)	16,008 (36.6%)	18,141 (37.5%)	18,353 (38.0%)	6,734 (38.3%)
75 and above	1,438 (16.9%)	2,651 (17.5%)	3,515 (19.3%)	4,973 (20.7%)	6,911 (21.7%)	9,071 (22.3%)	10,445 (22.8%)	10,504 (24%)	11,822 (24.4%)	12,698 (26.3%)	5,032 (28.6%)
Sex											
Male	4,517 (53.0%)	7,648 (50.6%)	9,163 (50.4%)	12,024 (50.0%)	15,844 (49.8%)	20,223 (49.7%)	22,580 (49.3%)	21,664 (49.5%)	24,201 (50.0%)	24,142 (50.0%)	8,708 (49.6%)
Female	3,999 (47.0%)	7,460 (49.4%)	9,030 (49.6%)	12,018 (50.0%)	15,994 (50.2%)	20,488 (50.3%)	23,183 (50.7%)	22,116 (50.5%)	24,227 (50.0%)	24,181 (50.0%)	8,857 (50.4%)
Marital status											
Married or Partnered	7,722 (90.7%)	14,001 (92.7%)	17,006 (93.5%)	22,635 (94.1%)	28,450 (89.4%)	37,521 (92.2%)	42,707 (93.3%)	41,022 (93.7%)	45,427 (93.8%)	45,188 (93.5%)	16,330 (93.0%)
Unmarried and Others	788 (9.3%)	1,098 (7.3%)	1,187 (6.5%)	1,407 (5.9%)	3,388 (10.6%)	3,190 (7.8%)	3,056 (6.7%)	2,758 (6.3%)	3,000 (6.2%)	3,134 (6.5%)	1,235 (7.0%)
Residence status											
Rural	1,737 (20.4%)	3,811 (25.2%)	4,730 (26.0%)	6,893 (28.7%)	9,416 (29.6%)	12,036 (29.6%)	13,970 (30.5%)	14,103 (32.2%)	15,375 (31.7%)	15,426 (31.9%)	5,699 (32.4%)
Urban	6,779 (79.6%)	11,296 (74.8%)	13,457 (74.0%)	17,138 (71.3%)	22,418 (70.4%)	28,673 (70.4%)	31,793 (69.5%)	29,677 (67.8%)	33,053 (68.3%)	32,897 (68.1%)	11,866 (67.6%)
Health insurance											
UEBMI	4,755 (55.8%)	8,305 (55.0%)	9,849 (54.2%)	12,523 (52.5%)	16,013 (50.7%)	20,728 (51.4%)	22,803 (50.3%)	21,209 (48.9%)	24,127 (50.1%)	24,592 (51.0%)	9,167 (52.3%)
URRBMI	2,726 (32.0%)	5,396 (35.7%)	6,679 (36.7%)	9,333 (39.1%)	12,807 (40.6%)	16,065 (39.8%)	18,611 (41.1%)	18,912 (43.6%)	20,855 (43.3%)	20,533 (42.6%)	7,354 (42.0%)
Others	1,035 (12.2%)	1,398 (9.3%)	1,654 (9.1%)	2,019 (8.5%)	2,747 (8.7%)	3,554 (8.8%)	3,884 (8.6%)	3,210 (7.4%)	3,142 (6.5%)	3,069 (6.4%)	1,001 (5.7%)

sd, standard deviation; T2DM, type 2 diabetes mellitus; UEBMI, urban employee basic medical insurance; URRBMI, urban and rural resident basic medical insurance.

### Prevalence of T2DM-related complications

Table 2 shows the prevalence of T2DM-related complications between 2013 and 2023. Throughout the study period, the prevalence of T2DM-related complications increased significantly, increasing from 30.4% in 2013 to 53.1% in 2023. Correspondingly, the average total CCI score rose steadily, from 5.2 (±2.9) in 2013 to 13.4 (±7.2) in 2023. Additionally, the mean complication counts progressively increased from 0.5 (±0.9) to 1.3 (±1.6), along with the complication-specific CCI score, which rose from 1.0 (±1.8) to 2.6 (±3.3). Ophthalmic, neurological, and circulatory complications were among the most reported and each exhibited a significant upward trend over the study period. Specifically, from 2013 to 2023, ophthalmic complications increased markedly from 14.1% to 30.8%, neurological complications from 17.7% to 37.6%, and circulatory complications from 5.7% to 27.0%.

**Table 2:** Prevalence of T2DM-related complications (2013–2023) [*n* (%)/mean (sd)].

Variables	2013	2014	2015	2016	2017	2018	2019	2020	2021	2022	2023
Number of patients with T2DM (*n*)	8,516	15,108	18,193	24,042	31,838	40,711	45,763	43,780	48,428	48,323	17,565
Prevalence of T2DM complications [*n* (%)]	2,591 (30.4%)	4,620 (30.6%)	5,983 (32.9%)	8,337 (34.7%)	11,673 (36.7%)	15,828 (38.9%)	19,036 (41.6%)	19,182 (43.8%)	23,149 (47.8%)	24,798 (51.3%)	9,325 (53.1%)
Total count [mean (sd)]	4.4 (2.2)	4.8 (2.7)	5.3 (3.1)	5.9 (3.4)	6.5 (3.7)	7.2 (4.0)	8 (4.3)	8.7 (4.7)	9.5 (4.9)	10.3 (5.2)	10.9 (5.5)
Total CCI [mean (sd)]	5.2 (2.9)	5.8 (3.5)	6.4 (4.0)	7.1 (4.4)	7.9 (4.8)	8.7 (5.3)	9.7 (5.7)	10.6 (6.2)	11.6 (6.5)	12.7 (6.9)	13.4 (7.2)
Complication count [mean (sd)]	0.5 (0.9)	0.6 (1.0)	0.6 (1.1)	0.7 (1.1)	0.7 (1.2)	0.8 (1.3)	0.9 (1.4)	1.0 (1.5)	1.1 (1.5)	1.2 (1.6)	1.3 (1.6)
Complication CCI [mean (sd)]	1.0 (1.8)	1.1 (2.0)	1.2 (2.1)	1.3 (2.2)	1.5 (2.4)	1.6 (2.6)	1.8 (2.8)	2.0 (2.9)	2.2 (3.0)	2.4 (3.2)	2.6 (3.3)
Prevalence of various complications [*n* (%)]											
T2DM with coma	14 (0.2%)	18 (0.1%)	11 (0.1%)	15 (0.1%)	24 (0.1%)	26 (0.1%)	21 (0.0%)	14 (0.0%)	10 (0.0%)	3 (0.0%)	0 (0.0%)
T2DM with ketoacidosis	200 (2.3%)	245 (1.6%)	245 (1.3%)	302 (1.3%)	351 (1.1%)	347 (0.9%)	300 (0.7%)	240 (0.5%)	157 (0.3%)	32 (0.1%)	0 (0.0%)
T2DM with kidney complications	841 (9.9%)	1,535 (10.2%)	1,977 (10.9%)	2,705 (11.3%)	3,967 (12.5%)	5,663 (13.9%)	7,247 (15.8%)	7,605 (17.4%)	8,941 (18.5%)	9,590 (19.8%)	3,772 (21.5%)
T2DM with ophthalmic complications	1,202 (14.1%)	2,183 (14.4%)	3,042 (16.7%)	4,116 (17.1%)	5,761 (18.1%)	8,127 (20.0%)	10,287 (22.5%)	10,629 (24.3%)	12,814 (26.5%)	13,698 (28.3%)	5,402 (30.8%)
T2DM with neurological complications	1,509 (17.7%)	2,773 (18.4%)	3,561 (19.6%)	5,283 (22.0%)	7,502 (23.6%)	11,101 (27.3%)	13,797 (30.1%)	14,131 (32.3%)	17,032 (35.2%)	17,656 (36.5%)	6,611 (37.6%)
T2DM with circulatory complications	485 (5.7%)	1,293 (8.6%)	1,902 (10.5%)	3,093 (12.9%)	4,892 (15.4%)	6,848 (16.8%)	8,884 (19.4%)	9,323 (21.3%)	11,538 (23.8%)	12,563 (26.0%)	4,736 (27.0%)
T2DM with other specified complications	143 (1.7%)	267 (1.8%)	355 (2.0%)	593 (2.5%)	873 (2.7%)	1,097 (2.7%)	1,342 (2.9%)	1,695 (3.9%)	3,495 (7.2%)	5,569 (11.5%)	2,390 (13.6%)

CCI, Charlson comorbidity index; sd, standard deviation; T2DM, type 2 diabetes mellitus.

### Complication onset and progression trajectory

Table 3 presents the median duration from the initial T2DM diagnosis to the onset of various T2DM-related complications. Among the 9,194 patients who developed any complication, the median time to onset was 7.5 years. The time to onset of complications varied across complication types. The longest median duration was observed in patients who developed T2DM with coma (8.7 years), followed by circulatory complications (8.0 years) and ophthalmic complications (7.9 years). In contrast, patients with ketoacidosis experienced complications earlier, with a median duration of 7.3 years. The median onset times of kidney, neurological, and other specified complications were 7.6, 7.7, and 7.5 years, respectively.

**Table 3:** Median duration from T2DM diagnosis to complication onset.

Type of diabetic complication	ICD-10 code	Number of patients	Time (median years)
Any type of diabetic complication	——	9,194	7.5
T2DM with coma (including hyperosmolar coma, hypoglycemia, and diabetic ketoacidosis)	E11.0, E14.0	156	8.7
T2DM with ketoacidosis (including diabetic ketoacidosis, lactic acidosis, and ketosis)	E11.1, E14.1	1,031	7.3
T2DM with kidney complications (e.g., diabetic nephropathy)	E11.2, E14.2	2,353	7.6
T2DM with ophthalmic complications (e.g., retinopathy, cataract, iritis)	E11.3, E14.3	3,448	7.9
T2DM with neurological complications (e.g., peripheral neuropathy, autonomic neuropathy, neuritis, neurogenic bladder, muscular atrophy)	E11.4, E14.4	5,352	7.7
T2DM with circulatory complications (e.g., peripheral vascular disease, cardiomyopathy, diabetic foot, ulcers, gangrene)	E11.5, E14.5	3,590	8.0
T2DM with other specified complications (e.g., arthropathy, neuropathic arthropathy, dermatopathy)	E11.6, E14.6	1,256	7.5

ICD-10, international statistical classification of diseases and related health problems, 10th revision; T2DM, type 2 diabetes mellitus.

Table 4 illustrates the trajectory of T2DM-related complications over one, five, and nine years based on the top ten most common complication combinations. At baseline (Year 0), 28,267 patients were complications-free. By Year 1, 74.7% of the patients remained complication-free, whereas 25.3% developed at least one complication. The most common first complication was neurological (4.3%), followed by ophthalmic (3.6%), kidney (2.1%), and circulatory (2.0%) complications. By Year 5, only 33.3% of patients remained without complications. The most frequent complication combinations were neurological only (11.1%), neurological and circulatory (7.3%), and neurological and ophthalmic complications (7.2%). At Year 9, the complexity of the combinations increased, with most patients experiencing multiple complications. The most common combinations were neurological (12.2%), followed by neurological and circulatory (11.0%), neurological, ophthalmic, and circulatory (8.4%), and neurological, kidney, ophthalmic, and circulatory complications (7.7%).

**Table 4:** Trajectories of T2DM-related complications over one, five, and nine years.

Top 10 combinations	Year 0			Year 1			Year 5			Year 9	
1	E1x.9	28,267 (100%)	→	E1x.9	21,128 (74.7%)	→	E1x.9	5,176 (33.3%)	→	E1x.4	368 (12.2%)
2				E1x.4	1,219 (4.3%)		E1x.4	1,723 (11.1%)		E1x.4, E1x.5	333 (11.0%)
3				E1x.3	1,014 (3.6%)		E1x.4, E1x.5	1,141 (7.3%)		E1x.3, E1x.4, E1x.5	254 (8.4%)
4				E1x.2	606 (2.1%)		E1x.3	1,122 (7.2%)		E1x.2, E1x.3, E1x.4, E1x.5	232 (7.7%)
5				E1x.5	580 (2.0%)		E1x.5	754 (4.8%)		E1x.3	211 (7.0%)
6				E1x.6	481 (1.7%)		E1x.3, E1x.4, E1x.5	745 (4.8%)		E1x.3, E1x.4	172 (5.7%)
7				E1x.1	311 (1.1%)		E1x.2	738 (4.7%)		E1x.9	165 (5.5%)
8				E1x.4, E1x.5	287 (1.0%)		E1x.3, E1x.4	634 (4.1%)		E1x.6	156 (5.2%)
9				E1x.5, E1x.4	209 (0.7%)		E1x.6	504 (3.2%)		E1x.2	150 (5.0%)
10				E1x.4, E1x.3	206 (0.7%)		E1x.2, E1x.3, E1x.4, E1x.5	469 (3.0%)		E1x.2, E1x.4, E1x.5	139 (4.6%)

T2DM, type 2 diabetes mellitus; E1x.9, T2DM without complications; E1x.0, T2DM with coma; E1x.1, T2DM with ketoacidosis; E1x.2, T2DM with kidney complications; E1x.3, T2DM with ophthalmic complications; E1x.4, T2DM with neurological complications; E1x.5, T2DM with circulatory complications; E1x.6, T2DM with other specified complications.

**Figure 1:**
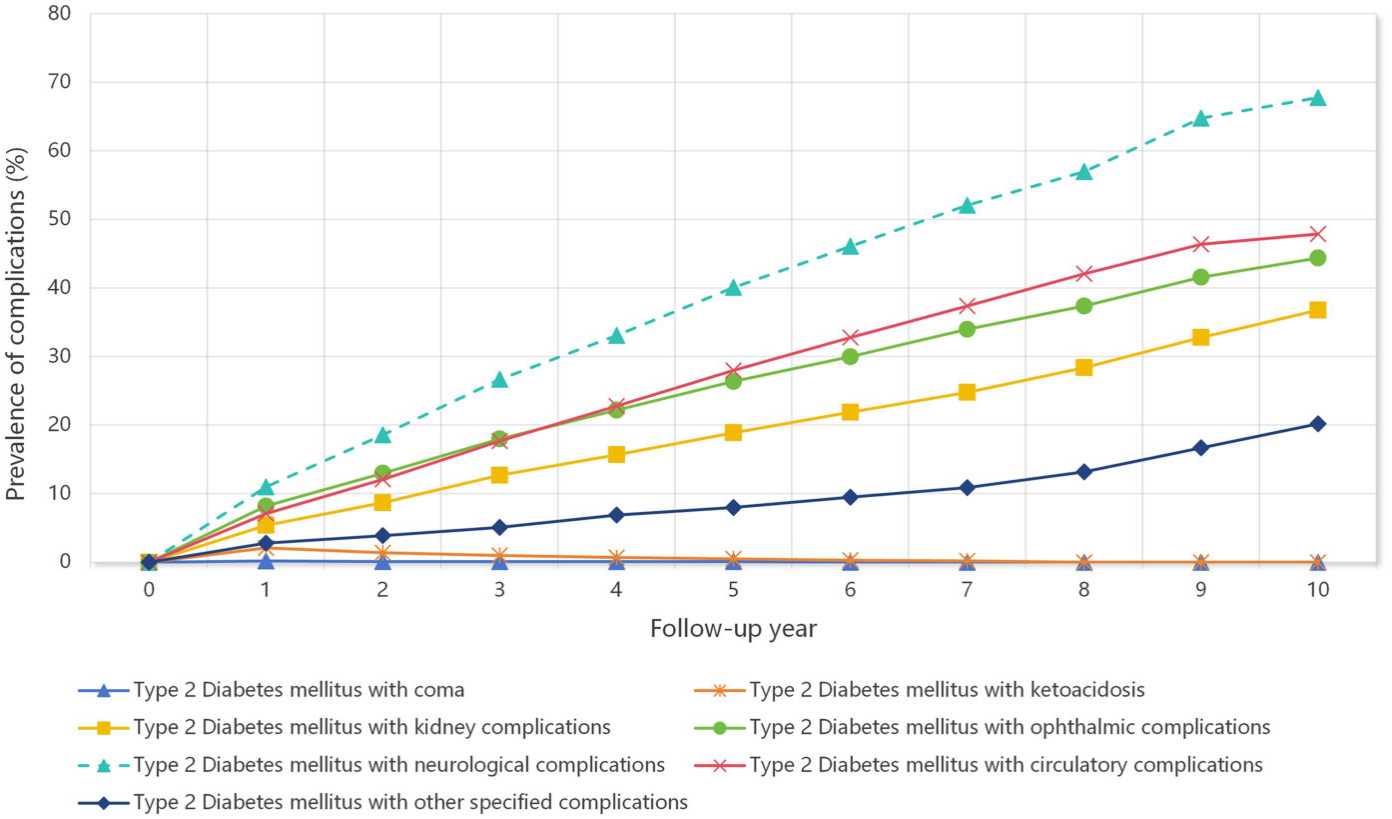
Prevalence trajectories of type 2 diabetes mellitus-related complications over a ten-year follow-up period.

Figure 1 shows the prevalence trajectories of different T2DM-related complications over a ten-year follow-up period. Among all complication types, neurological complications showed the most pronounced increase, with the prevalence rising steadily from 0% at baseline to nearly 70% by Year 10. Circulatory and ophthalmic complications also substantially increased, reaching approximately 47.9% and 44.4%, respectively, by the end of the observation period. Kidney complications showed a similar increasing trend, with a prevalence of 36.8% by Year 10. The prevalence of other specified complications increased gradually to 20.2%, whereas coma and ketoacidosis remained rare throughout the study period with minimal prevalence changes.

### Factors associated with the occurrence of T2DM-related complications

**Figure 2:**
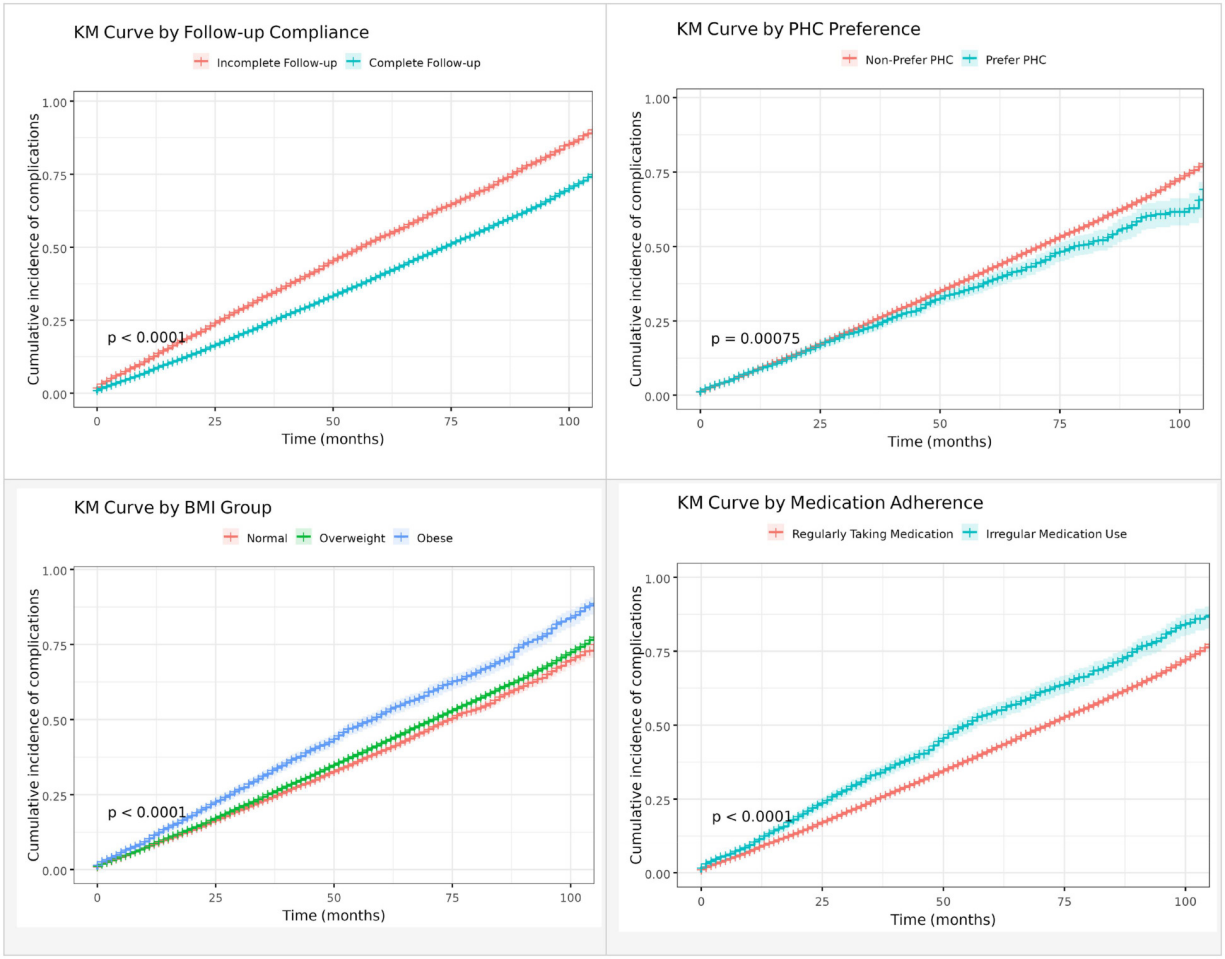
Kaplan–Meier curves for the occurrence of type 2 diabetes mellitus-related complications based on follow-up compliance, primary health care preference, body mass index and medication adherence.

Figure 2 presents Kaplan–Meier (KM) curves illustrating the cumulative incidence of type 2 diabetes mellitus (T2DM)-related complications stratified by follow-up compliance, primary health care (PHC) preference, body mass index (BMI), and medication adherence. Patients with complete follow-up (≥4 visits per year) showed significantly lower complication rates compared to those with incomplete follow-up (log-rank test, *p* < 0.0001). Similarly, individuals who preferred PHC services (PHC visits ≥50%) experienced a lower incidence of complications than those who did not (log-rank test, *p* = 0.00075). In terms of BMI, both overweight (24 ≤ BMI < 28) and obese (BMI ≥ 28) patients showed a higher cumulative incidence of complications compared with those with normal BMI (log-rank test, *p* < 0.0001). Moreover, regular medication adherence was associated with a substantially reduced risk of complications compared to irregular medication use (log-rank test, *p* < 0.0001).

Table 5 summarizes the factors associated with the occurrence of T2DM-related chronic complications based on the Cox regression analysis. Patients with ≥4 follow-up visits per year had a 37% lower risk (HR = 0.63, *p* < 0.001), and those who preferred PHC had a 15% lower risk (HR = 0.85, *p* < 0.001) compared with their counterparts. The risk of complications was 17% higher among drinkers (HR = 1.17, *p* < 0.001), 29% higher among overweight individuals (24 ≤ BMI < 28) (HR = 1.29, *p* < 0.001), and 39% higher among obese individuals (BMI ≥ 28) (HR = 1.39, *p* < 0.001). Patients with irregular medication use had a 21% higher risk (HR = 1.21, *p* < 0.001). Urban residents showed a significantly lower risk compared with rural residents (HR = 0.92, *p* < 0.001). In addition, age group, marital status, and health insurance type were also significantly associated with the risk of developing T2DM-related chronic complications.

**Table 5:** Factors influencing the occurrence of T2DM-related chronic complications (Cox regression analysis).

Variable	HR (95% CI)	*P* value
Follow-up visits ≥4 times/year (ref: <4 times/year)	0.63 (0.61, 0.65)	<0.001
Prefer PHC (ref: PHC visit <50%)	0.85 (0.79, 0.91)	<0.001
Age (ref:45–54)		
55–64	0.89 (0.86, 0.93)	<0.001
65–74	0.89 (0.86, 0.92)	<0.001
>75	0.90 (0.86, 0.94)	<0.001
Female (ref: Male)	1.00 (0.98, 1.03)	0.717
Unmarried and Others (ref: Married or Partnered)	1.09 (1.05, 1.14)	<0.001
Urban (ref: Rural)	0.92 (0.89, 0.94)	<0.001
Current or Former Smoker (ref: Non-Smoker)	1.07 (0.97, 1.17)	0.171
Drinker (ref: Non-Drinker)	1.17 (1.11, 1.22)	<0.001
BMI (ref: BMI < 24)		
Overweight (24 ≤ BMI < 28)	1.29 (1.24, 1.33)	<0.001
Obesity (BMI > 28)	1.39 (1.30, 1.47)	<0.001
Irregular Medication Use (ref: Regular Use)	1.21 (1.13, 1.29)	<0.001
Health insurance (ref: UEBMI)		
URRBMI	0.92 (0.90, 0.95)	<0.001
Other	0.91 (0.87, 0.96)	<0.001
Total CCI score	1.00 (0.99, 1.01)	0.992

CCI, Charlson comorbidity index; CI, confidence interval; HR, hazard ratio; PHC, primary health care; T2DM, type 2 diabetes mellitus; UEBMI, urban employee basic medical insurance; URRBMI, urban and rural resident basic medical insurance.

HRs represent the relative risk of diabetes-associated complications. HRs >1 and <1 indicate increased and decreased risk, respectively. The sample size for the Cox regression analysis was 91,428.

## Discussion

T2DM has emerged as a significant public health concern in China, and its prevalence has escalated rapidly in recent decades. This study provides a comprehensive overview of the burden, progression, and factors associated with the occurrence of T2DM-related complications in Shandong province in China based on an extensive, integrated dataset compiled from multiple administrative and clinical sources. We observed a clear upward trend in the prevalence and complexity of complications over time, with neurological, ophthalmic, and circulatory conditions emerging early and commonly. Notably, several modifiable factors are associated with the risk of complications, highlighting the importance of timely follow-up, primary care engagement, and consistent medication adherence in reducing long-term adverse outcomes.

First, we identified a substantial increase in the prevalence of T2DM-related complications over ten years, from 30.4% in 2013 to 53.1% in 2023. Among these, ophthalmic, neurological, and circulatory complications were the most common, each showing marked upward trends: ophthalmic complications increased from 14.1% to 30.8%; neurological complications increased from 17.7% to 37.6%; and circulatory complications increased from 5.7% to 27.0%. Consistent with global evidence, the T2DM burden extends beyond hyperglycemia, as it is frequently accompanied by serious complications. Previous studies have indicated that among Chinese individuals with diabetes, approximately 14.9% also suffer from cardiovascular diseases (36), 21.3% have chronic kidney disease (37), 16.3% experience diabetic retinopathy, and 3.2% have vision-threatening diabetic retinopathy (16). These complications become increasingly prevalent and severe with a longer duration of diabetes and poorer metabolic control, including elevated glucose and lipid concentrations, as well as higher blood pressure, subsequently leading to increased mortality rates and escalating healthcare expenditures (38).

Second, our study provides new insights by revealing distinct trajectories and timings of complication onset that is consistent with prior work emphasizing the importance of early clustering in certain conditions (9). The median time from T2DM diagnosis to the onset of complications was 7.5 years, with notable differences across complication types. Neurological, ophthalmic, and circulatory complications occurred relatively early in the disease course, which echoes the findings of Martinez-De la Torre et al., who reported that specific comorbidity clusters tend to emerge early in the disease course (10). This study also analyzed how combinations of complications evolve throughout the disease course, finding that they become increasingly complex over time, with most patients developing multiple complications by Year 9. The most common patterns was neurological complications alone (12.2%), followed by combinations involving circulatory (11.0%), ophthalmic (8.4%), and kidney complications (7.7%). The growing complexity of comorbid profiles, particularly in Years 5 and 9, underscores the urgent need for personalized and continuous care strategies. Personalized diabetes management, which tailors treatment plans to individual patient characteristics such as age, comorbidities, and lifestyle, improves glycemic control and reduce the risk of complications (39). For instance, the American Diabetes Association and the European Association for the Study of Diabetes advocate individualized glycemic targets to enhance patient outcomes and minimize adverse effects (40). In addition, integrated personalized diabetes management approaches that combine interdisciplinary care and digital technologies have demonstrated promise in addressing the multifaceted needs of patients with T2DM (41).

We also focused on the factors associated with the risk of developing T2DM-related complications. Notably, the association of behavioral and healthcare utilization factors with complication risk provides additional value beyond the clinical variables alone. Our analysis highlights the crucial role of regular follow-ups and PHC use, which are associated with slower progression of T2DM-related complications.

The NEPHSP was designed to enhance early diagnosis, standardize chronic disease management, and reduce complication risks by integrating essential services such as health education, lifestyle counseling, and treatment adherence monitoring into community-based care (42). However, its implementation has been unequal, particularly in rural and underserved areas (42, 43). Within this context, our study highlights the protective effects of more intensive follow-up (≥4 visits per year) and a preference for PHC on the risk of complications—findings that align with the core objectives of the NEPHSP while also emphasizing the need for strengthened implementation and equitable service delivery (24, 44).

More intensive follow-up may enable the early detection of metabolic abnormalities such as elevated blood glucose and blood pressure, allowing for timely intervention and treatment plan adjustment (45). It also reinforces patient education and self-management behaviors, which are essential for long-term glycemic control and complication prevention (46). Moreover, consistent follow-up supports personalized treatment strategies tailored to the evolving clinical needs of patients, thereby enhancing the effectiveness of chronic disease management (40). Additionally, frequent contact with healthcare providers fosters better patient–provider communication and trust, which has been shown to improve adherence to treatment and medical advice (47, 48).

This study also revealed the value of a PHC-centered approach in reducing the burden associated with T2DM-related complications in China. First, PHC facilities in China are attempting to adopt integrated care models that combine medical treatment with preventive services, enabling early detection and intervention for diabetes-related risks (49–51). Second, PHC centers are more accessible than hospitals, especially in rural and underserved areas, and provide continuous and coordinated care essential for long-term disease control (52, 53). Third, managing diabetes through PHC is more cost-effective than hospital-based care, thereby reducing patients’ financial burdens and improving treatment adherence, which are critical for preventing complications (54). Finally, implementing of electronic health records and improving health information systems at the PHC level enhances the tracking of patient conditions and supports timely, data-driven interventions (55).

These findings suggest that strengthening the delivery and uptake of NEPHSP services, particularly through the improved integration of PHC and hospital-based care, better training for community health workers, and incentives for adherence, could be pivotal in delaying or preventing the onset of diabetic complications. Promoting PHC-centered models and leveraging routinely collected health data for monitoring and feedback may further enhance the effectiveness and efficiency of chronic disease management in China.

Notably, although rural patients in our study had more frequent primary health care follow-up visits (Supplementary Table S3), they still exhibited higher hazard ratios for diabetes complications. One plausible explanation is that rural primary care services face limitations in healthcare quality and resources that undermine effective diabetes management. Studies in China have noted that community-based diabetes care in rural areas achieves poorer outcomes, with significantly lower rates of diabetes awareness, treatment adherence, and glycemic control—and less comprehensive complication screening—compared to urban areas (56). Furthermore, structural barriers in rural regions—such as limited access to specialists, diabetes education programs, and advanced monitoring or treatment options—can lead to delays in identifying and addressing complications. This aligns with broader evidence: a large Chinese cohort reported that while diabetes is more prevalent in cities, its excess mortality impact is greater in rural areas (57). Together, these findings underscore the need to improve the effectiveness of rural diabetes management in addition to simply increasing its frequency.

This study makes several novel contributions to the existing literature on T2DM complications. First, it leverages an integrated, population-based cohort of over five million individuals from the Cheeloo LEAD, enabling real-world and long-term tracking of complication trajectories across diverse demographic and healthcare contexts. Second, it is among the first studies to incorporate PHC utilization—measured by follow-up frequency and PHC preference—into trajectory and survival analyses, providing new empirical evidence on how routine follow-up and PHC engagement are associated with the onset and progression of complications. Third, unlike prior studies that focused on single complication types, this research comprehensively depicts the ten-year temporal evolution and clustering patterns of multiple diabetes-related complications. Together, these innovations extend the understanding of how behavioral and healthcare service factors interact with disease progression, offering valuable insights for strengthening PHC-based chronic disease management in China and other LMIC settings.

This study has several limitations. First, although our data source integrated multiple administrative and clinical records, it lacked key variables that reflected the patients’ socioeconomic status, such as income, education level, and occupation. These factors are known to be associated with access to care, treatment adherence, and disease progression, and their absence limits our ability to fully account for the social factors associated with T2DM-related complications. Although we used place of residence (urban/rural) as a proxy indicator and observed significant disparities, we acknowledge that these proxies may not fully capture the complexity of social determinants of health, and this limitation should be considered when interpreting the results. Second, the diagnoses relied on electronic medical records and ICD-10 codes, which may have been subject to underreporting or misclassification. Third, patient engagement and data completeness may affect behavioral measures, such as follow-up frequency and medication adherence. Furthermore, as complication data were primarily obtained from inpatient records, individuals who did not require hospitalization were excluded. This may have resulted in a higher-risk cohort and an overestimation of complication incidence. However, the observed protective effect of PHC in this high-risk population highlights its potential in delaying complications. Additionally, due to the lack of death records in the dataset, we were unable to account for death as a competing risk, which may have biased the estimation of complication incidence. Future studies incorporating detailed socioeconomic data, non-hospitalized patients, and mortality information would provide a more comprehensive understanding of disparities and outcomes in T2DM.

Although this study utilized a large population-based dataset from Shandong Province, we acknowledge that the generalizability of our findings to the entire Chinese population or other countries is limited. Shandong, with a population exceeding 101.5 million (over 100 million), is the second most populous province in China. It encompasses a wide spectrum of urban and rural communities and reflects considerable heterogeneity in economic development across its jurisdictions. For instance, in 2024, the per capita GDP of Qingdao, a coastal and economically developed city, reached 22,459.50 USD, whereas that of Liaocheng, an inland and less developed city, was only around 5,300 USD. This substantial regional disparity within Shandong mirrors the broader developmental gaps observed across China and many global contexts. Therefore, while caution is warranted, the demographic, economic, and healthcare diversity within Shandong allows for a certain degree of analogy to both other Chinese provinces and low- and middle-income settings globally. Additionally, Shandong's moderate socioeconomic status and relatively mature health information infrastructure position it as a valuable sentinel region for understanding chronic disease patterns in rapidly transitioning eastern and central China. Nonetheless, given the marked differences in demographic structure, healthcare capacity, and the implementation of essential public health services across provinces, further studies from western or underdeveloped areas are still needed to validate the consistency and broader applicability of these findings. Moreover, the analytical framework and key insights of this study may offer useful implications for other low- and middle-income countries with similar primary healthcare systems, contributing to global efforts to improve chronic disease management.

In conclusion, patients with T2DM face a substantial and growing burden of complications, with most complications developing within ten years of diagnosis. Neurological, ophthalmic, and circulatory complications have emerged as the most common and earliest complications, often in combination, underscoring the need for early and continuous monitoring. A high follow-up frequency, preference for PHC services, and regular medication adherence were associated with a significantly lower complication risk, whereas an overweight status, alcohol consumption, and irregular treatment behaviors were associated with an increased risk. These findings strongly support integrated community-based and behaviorally informed chronic disease management strategies. Tailored prevention and management strategies, particularly in the early years after diagnosis, are essential for reducing the risk of long-term complication and promoting better health outcomes in patients with T2DM in low- and middle-income settings.

## Data Availability

The data analyzed in this study is subject to the following licenses/restrictions: the individual-level data used in this study are part of the Cheeloo Lifespan Electronic Health Research Data Library (Cheeloo LEAD) and cannot be shared publicly due to institutional regulations and data use agreements. Access to the data requires prior approval from the data management committee and is restricted to qualified researchers. Researchers interested in accessing the Cheeloo LEAD database must submit a formal research proposal and obtain ethical approval from the Ethics Committee of Shandong University. Data access will only be granted upon meeting all institutional and ethical requirements. For inquiries regarding data access, please contact the School of Public Health, Shandong University (201999000066@sdu.edu.cn). Requests to access these datasets should be directed to School of Public Health, Shandong University (201999000066@sdu.edu.cn).
