# Supplementary material for: Complication development trajectories for patients with type 2 diabetes mellitus: evidence from a five-million retrospective cohort study
Source: Front Health Serv. 2025 Nov 25;5:1699513. doi: 10.3389/frhs.2025.1699513 (PMC12685925; doi:10.3389/frhs.2025.1699513)
Supplement: Supplementary file 1 [file Table1.docx]

**Complication Trajectories and Determinants among Patients with Type 2 Diabetes Mellitus: Evidence from a Population-Based Study in Shandong Province, China (2013–2023)**

*Supplementary file*


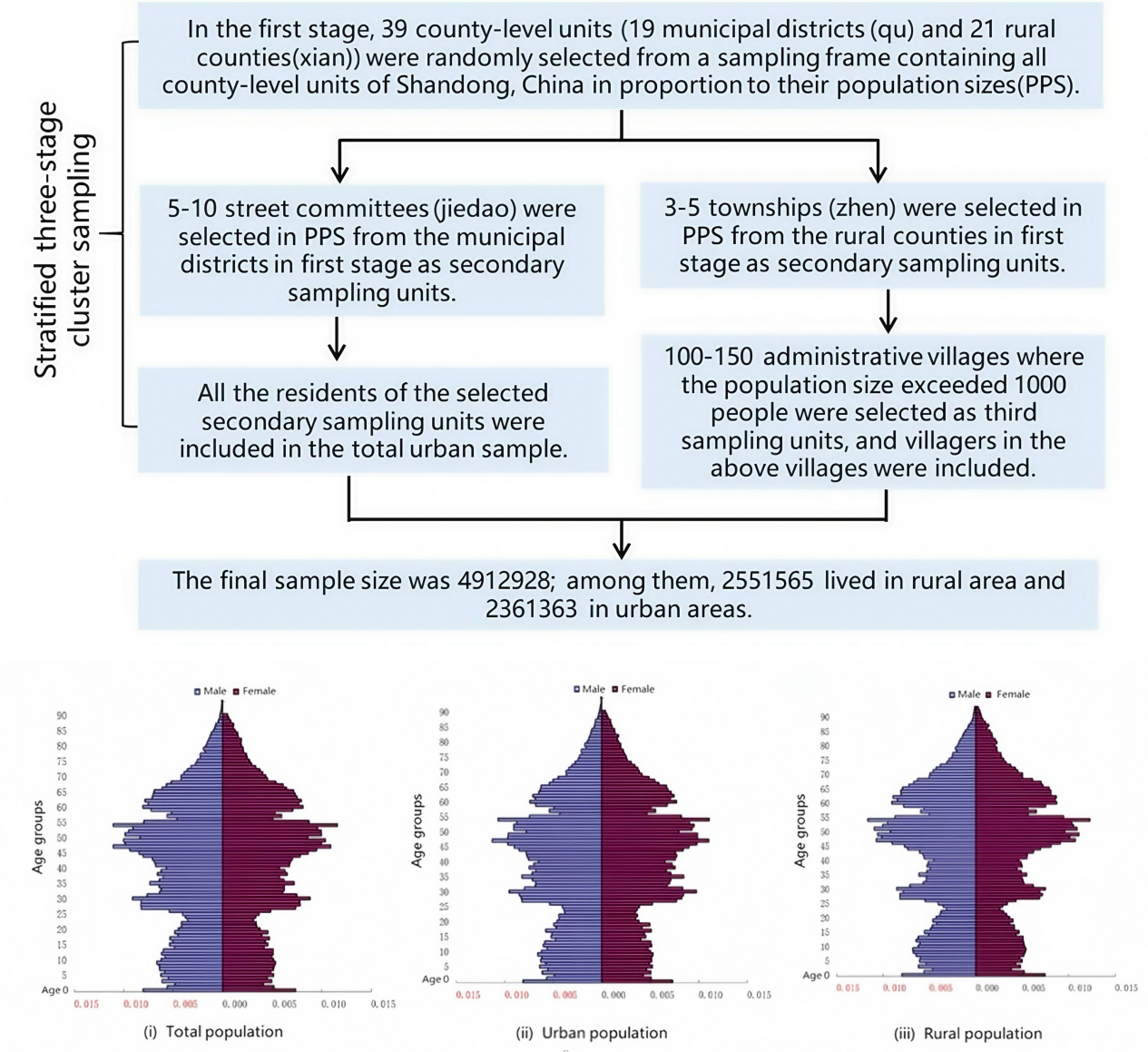


**Figure S1** Three-stage cluster sampling process and demographic characteristics of the sample population.

Note：The Cheeloo Lifespan Electronic Health Research Data Library (Cheeloo LEAD) originally included 4,912,928 individuals sampled from 39 counties (districts) in Shandong Province, China. After excluding residents without a diagnosis of type 2 diabetes mellitus (T2DM), those lacking hospitalization records or with missing key demographic or diagnostic information, and individuals with incomplete follow-up data or duplicate records, 122,236 patients with confirmed T2DM were included in the final analytic cohort.

**Table S1** Classification of T2DM–Related Complications, Corresponding ICD Codes, and CCI Scores

| Type of Complications | ICD code | CCI score |
| --- | --- | --- |
| T2DM without complications | E11.9, E14.9 (E1x.9) | 1 |
| T2DM with coma (including hyperosmolar coma, hypoglycemia, and diabetic ketoacidosis) | E11.0, E14.0 (E1x.0) | 2 |
| T2DM with ketoacidosis (including diabetic ketoacidosis, lactic acidosis, and ketosis) | E11.1, E14.1 (E1x.1) | 2 |
| T2DM with kidney complications (e.g., diabetic nephropathy) | E11.2, E14.2 (E1x.2) | 2 |
| T2DM with ophthalmic complications (e.g., retinopathy, cataract, iritis) | E11.3, E14.3 (E1x.3) | 2 |
| T2DM with neurological complications (e.g., peripheral neuropathy, autonomic neuropathy, neuritis, neurogenic bladder, muscular atrophy) | E11.4, E14.4 (E1x.4) | 2 |
| T2DM with circulatory complications (e.g., peripheral vascular disease, cardiomyopathy, diabetic foot, ulcers, gangrene) | E11.5, E14.5 (E1x.5) | 2 |
| T2DM with other specified complications (e.g., arthropathy, neuropathic arthropathy, dermatopathy) | E11.6, E14.6 (E1x.6) | 2 |

Note: CCI= Charlson Comorbidity Index;

**Table S2** Demographic Characteristics of Patients with T2DM at Baseline [n(%) /mean(sd)]

| Variables | Demographic characteristics |
| --- | --- |
| Total | 122236 |
| Time | 2013-2023 |
| Age (years) | 64.4 (10.0) |
| 45-54 | 22845 (18.7%) |
| 55-64 | 38871 (31.8%) |
| 65-74 | 40161 (32.8%) |
| 75 and above | 20542 (16.8%) |
| Sex |  |
| Male | 60532 (49.4%) |
| Female | 61887 (50.6%) |
| Marital status |  |
| Married or Partnered | 112845 (92.2%) |
| Unmarried and Others | 9561 (7.8%) |
| Residence status |  |
| Rural | 39947 (32.6%) |
| Urban | 82460 (67.4%) |
| Health insurance |  |
| UEBMI | 55652 (45.8%) |
| URRBMI | 55064 (45.3%) |
| Others | 10763 (8.9%) |

Note: UEBMI=Urban employee basic medical insurance, URRBMI=Urban and rural resident basic medical insurance;

**Table S3** Annual Follow-Up Visits per Patient in Urban and Rural Areas, 2013–2023 [mean(sd)]

| Year | Urban | Rural | P value |
| --- | --- | --- | --- |
| 2013 | 5.81 (1.63) | 6.86 (2.18) | <0.001 |
| 2014 | 5.79 (1.71) | 6.72 (2.37) | <0.001 |
| 2015 | 5.80 (1.76) | 6.66 (2.27) | <0.001 |
| 2016 | 5.75 (1.53) | 6.79 (2.69) | <0.001 |
| 2017 | 5.78 (1.81) | 6.84 (2.84) | <0.001 |
| 2018 | 5.77 (1.61) | 6.78 (2.38) | <0.001 |
| 2019 | 5.75 (1.61) | 6.80 (2.55) | <0.001 |
| 2020 | 5.75 (1.58) | 6.78 (2.58) | <0.001 |
| 2021 | 5.77 (1.68) | 6.75 (2.51) | <0.001 |
| 2022 | 5.79 (1.63) | 6.75 (2.59) | <0.001 |
| 2023 | 5.77 (1.71) | 6.77 (2.68) | <0.001 |
